# Opioid-Induced Regulation of Cortical Circular-*Grin2b*_011731 Is Associated with Regulation of *circGrin2b* Sponge Target *miR-26b-3p*

**DOI:** 10.3390/ijms26115010

**Published:** 2025-05-22

**Authors:** Aria Gillespie, Stephanie E. Daws

**Affiliations:** Daws Laboratory, Center for Substance Abuse Research, Department of Neural Sciences, Lewis Katz School of Medicine, Temple University, Philadelphia, PA 19140, USA

**Keywords:** morphine, opioids, splicing, circular RNA, microRNA

## Abstract

Opioid use induces neurobiological adaptations throughout mesolimbic brain regions, such as the orbitofrontal cortex (OFC), which mediates decision-making and emotional–cognitive regulation. Previously, we showed that a circular RNA (circRNA) species, rno_*circGrin2b*_011731 (*circGrin2b*), is upregulated in the OFC of rats following chronic self-administration (SA) of the opioid heroin. *circGrin2b* is derived from *Grin2b*, which encodes the regulatory subunit of the glutamate ionotropic NMDA receptor, GluN2B. However, the upstream regulatory mechanisms of *circGrin2b* biogenesis and the downstream consequences of *circGrin2b* dysregulation remain unknown. We hypothesized that opioid-induced elevation of *circGrin2b* is accompanied by regulation of circRNA biogenesis enzymes, and that *circGrin2b* may sponge microRNAs (miRNAs), as miRNA sponging is a well-described characteristic of circRNAs. To test these hypotheses, we established an in vitro primary cortical cell culture model to examine alterations in *circGrin2b* expression following exposure to the opioid morphine. We measured mRNA expression of known circRNA splicing factors and observed significant downregulation of Fused in Sarcoma (*Fus*), a negative regulator of circRNA biogenesis, following 90 min or 24 h of morphine exposure. Downregulation of *Fus* at 24 h post-morphine was accompanied by upregulation of *circGrin2b* and downregulation of *miR-26b-3p*, a predicted miRNA target of *circGrin2b*. Luciferase reporter assays confirmed interaction of *miR-26b-3p* with *circGrin2b*. Finally, we report a significant negative relationship between *circGrin2b* and *miR-26b-3p* expression in the OFC of rats following heroin SA. We conclude that regulation of *circGrin2b* is an opioid-induced neuroadaptation that may impact downstream signaling of miRNA pathways in the frontal cortex.

## 1. Introduction

Opioid Use Disorder (OUD) is a chronic relapsing neurological disorder affecting more than 2 million Americans annually; opioids are involved in two-thirds of all overdose deaths in the US [1,2,3,4]. Despite currently available pharmacological treatment methods, between 50–60% of patients experience recurrence of use within the first year of recovery, and achieving stable abstinence can take 5–10 years [5,6,7]. This prolonged recovery period is due to complex drug-induced neuroadaptations which persist in the absence of drug and mediate OUD phenotypes. These neuroadaptations drive drug-associated cue learning and memory, and compulsive drug-seeking behaviors [8,9,10]. Currently available pharmacotherapies work primarily at mu-opioid receptors (MOR), yet opioids induce additional neuroadaptations beyond the MOR that contribute to mechanisms underlying OUD [10,11,12,13,14,15]. Thus, further research is required to elucidate the mechanisms mediating opioid-induced neuroadaptations.

Circular RNAs (circRNAs) are highly conserved regulators of gene expression, neuroplasticity, and behavior [16,17,18,19]. Because of the myriad functions of circRNAs, they have recently emerged as mediators of molecular and behavioral phenotypes of neuropsychiatric disorders [20,21,22,23,24,25,26,27]. Unlike other species of non-coding RNAs, circRNAs do not have their own transcripts. Instead, circRNAs are derived from pre-mRNA and are generated via a back-splicing event during which the 3′ end of a downstream splice donor site attacks the 5′ end of an upstream splice acceptor site, resulting in covalently bound 5′–3′ tails, and a circular structure [18,28]. Interestingly, compared to other mammalian tissues, circRNAs are most highly expressed in the brain, particularly within the frontal cortex; this enrichment is likely due to intrinsic characteristics of neural genes that allow for circularization, namely a high abundance of Alu elements and long intronic sequences [16,29]. Early research into the function of circRNAs has implicated neuronally expressed circRNAs in epigenetic regulation of synaptic plasticity and cognition, due to the following reasons: 1. a majority of circRNAs expressed in the brain are derived from synaptic coding genes; 2. circRNAs are dynamically expressed both spatially and temporally, with increased localization in neuronal regions participating in synaptic transmission and upregulation during periods of neural development; and 3. circRNA abundance is highly correlated with brain complexity and cognitive function [16,24,30,31]. Consistent with these findings, recent research has found circRNA dysregulation, particularly within the frontal cortex, across several diverse neuropathologies affecting cognitive processes like neurodegenerative disorders such as Parkinson’s and Alzheimer’s Diseases, as well as substance use disorders (SUDs) [21,22,27,32,33,34,35,36,37]. SUD-induced circRNA dysregulation has been observed in both rodent models and human postmortem tissues [25,32,33,38,39,40,41,42,43]. Additionally, methamphetamine, morphine, heroin, cocaine, and alcohol have been found to regulate circRNAs within the prefrontal cortex (PFC) and/or orbitofrontal cortex (OFC), two regions of the frontal cortex heavily involved in SUD pathologies [34]. While circRNAs have emerged as key mediators in neuropsychiatric pathologies, the molecular mechanisms for the vast majority of circRNAs remain largely unknown. Despite this fact, the aforementioned studies highlight the importance of characterizing drug-induced regulation of circRNAs, as well as uncovering the unique contributions of drug-associated circRNAs to molecular and behavioral phenotypes that result from drug exposure.

In a prior study, we employed a preclinical rat model of voluntary self-administration (SA) of the opioid heroin to demonstrate that heroin dysregulates expression of circRNAs in the OFC [32], a key region of the rodent frontal cortex that sustains lasting motivation for opioids [44,45,46,47]. We identified upregulation of OFC *rno-circGrin2b-011731* (*circGrin2b*), a novel circular splice-variant of *Grin2b*, as a heroin-induced neuroadaptation. *Grin2b* encodes the glutamate ionotropic NMDA receptor- subunit 2B (GluN2B) protein. Identification of opioid-induced dysregulation of *circGrin2b* is especially relevant, as linear *Grin2b* and its protein product GluN2B are essential for formation of opioid-induced learning and memory [14,48,49,50,51]. However, the mechanism of opioid-induced dysregulation of *circGrin2b* and downstream consequences of *circGrin2b* signaling remain unknown.

To begin to address these gaps in knowledge, we demonstrated that morphine exposure dynamically regulates *circGrin2b* in primary frontal cortical cultures, establishing a model system to recapitulate our previously reported in vivo findings. Utilizing this in vitro model, we identified putative splicing factors regulated by morphine that may regulate circRNA biogenesis. Finally, we reported significant regulation of a candidate *circGrin2b* microRNA (miRNA) sponge-target following morphine exposure, *miR-26b-3p*, and demonstrated physical association between *miR-26b-3p* and a *circGrin2b* reporter. This study provides critical insight into the molecular consequences of opioid-induced dysregulation of *circGrin2b*, a novel splice variant formed from a gene essential for drug-induced neuropathology.

## 2. Results

### 2.1. The Splicing Enzyme Fus Is Regulated by Opioid Exposure

**Figure 1:**
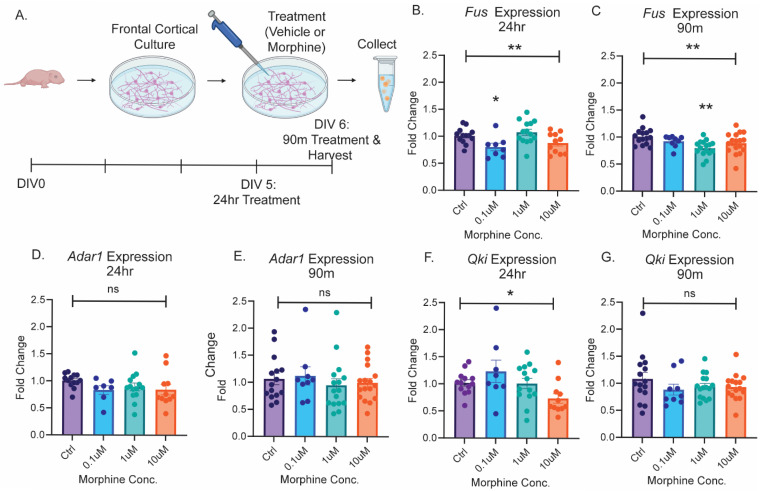
Morphine exposure regulates expression of enzymes involved in circRNA biogenesis. (**A**) Overview of experimental design and treatment of primary cortical cells with morphine. (**B**–**G**) Expression of putative circRNA biogenesis enzymes following 24 h morphine exposure (**B**,**D**,**F**) and 90 min (**C**,**E**,**G**) morphine exposure for *Fus* (**B**,**C**), *Adar1* (**D**,**E**), and *Qki* (**F**,**G**), Error bars indicate SEM. ** and * above the solid line indicate one-way ANOVA, *p* < 0.01 and *p* < 0.05, respectively. ** and * directly above histogram indicate *p* < 0.01 and *p* < 0.05 versus vehicle, respectively, for post hoc tests. ns, not significant.

Previously, we reported significant upregulation of 76 circRNAs in the OFC, a subregion of the frontal cortex, following 10 days of chronic heroin SA in adult male and female rats [32]. Upregulated circRNAs included *circGrin2b*, an exonic circRNA containing 599 nucleotides (nt) derived from exon 3 of *Grin2b* (Supplementary Figure S1). Although several enzymes have been linked to circRNA biogenesis, the circRNA substrates of each enzyme have not been fully described. Moreover, the splicing enzyme(s) responsible for *circGrin2b* biogenesis are currently unknown. To begin to elucidate the potential mechanisms regulating opioid-induced *circGrin2b* biogenesis, we examined the expression of three splicing factors known to be involved in circRNA splicing [52,53,54,55,56], in cortical cultures at two timepoints following exposure to three different concentrations of the opioid morphine. Primary frontal cortex cells were isolated from post-natal day one (PN1) rat pups and cultured for five days prior to treatment. Cortical cultures were treated with 0.1 µM, 1µM, or 10 µM morphine, or vehicle, for 24 h, or 90 min. 24 h cortical cultures were treated on DIV5, and 90 min cortical cultures were treated on DIV 6 (Figure 1A). Following cell harvest, mRNA expression of Fused in Sarcoma (*Fus*), Adenosine deaminase acting on RNA 1 (*Adar1*), and Quaking (*Qki*) were measured with qPCR. *Fus* was significantly downregulated in primary cortical cultures following both 24 h and 90 min of morphine exposure (one-way ANOVA 24 h: F (3, 42) = 4.877, *p* = 0.0053; Kruskal–Wallis 90 min: *p* = 0.0072; Figure 1B,C). Post hoc tests revealed significant downregulation of *Fus* in 24 h 0.1 µM and 90 min 1 µM treated cortical cultures compared to vehicle (Dunnett’s post hoc test: 24 h 0.1 µM *p* = 0.0279; Dunn’s post hoc test: 90 min 1 µM *p* = 0.0037; Figure 1B,C). Morphine treatment did not significantly alter the expression of *Adar1* at either 24 h or 90 min timepoints (Kruskal–Wallis 24 h: *p* = 0.1034; one-way ANOVA 90 min: F (3, 53) = 0.6047, *p* = 0.6149; Figure 1D,E). *Qki* expression was significantly altered following 24 h morphine exposure but not 90 min (Kruskal–Wallis, 24 h: *p* = 0.0500; 90 min: *p* = 0.6137; Figure 1F,G). However, post hoc tests did not reveal significant differences between vehicle and morphine treated groups for *Qki* after 24 h morphine exposure. We conclude that acute morphine exposure can significantly downregulate expression of *Fus*, a potential negative regulator of circRNA biogenesis [56].

### 2.2. Opioid-Induced Regulation of circGrin2b In Vitro Corresponds to Regulation of Putative circRNA Biogenesis Enzyme Fus

**Figure 2:**
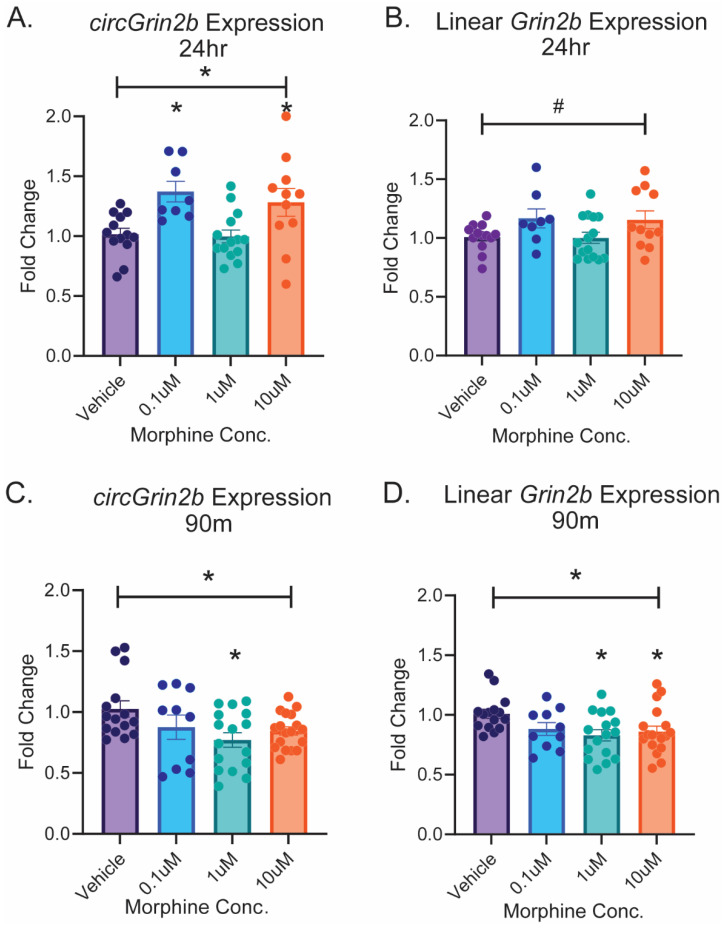
Morphine exposure regulates expression of *circGrin2b* in primary cortical cultures. (**A**–**D**) Expression of *circGrin2b* (**A**,**C**) and linear *Grin2b* (**B**,**D**) at 24 h (**A**,**B**), or 90 min (**C**,**D**) after morphine exposure. Error bars indicate the standard error of the mean (SEM). * above the solid line indicates one-way ANOVA, *p* < 0.05. # above the solid line denotes one-way ANOVA *p* < 0.1 but *p* > 0.05; * directly above histogram indicate *p* < 0.05 versus vehicle post hoc test.

To characterize *circGrin2b* regulatory mechanisms and downstream consequences in vitro, we sought to first identify a timepoint and morphine dose in primary cortical cultures that may recapitulate our previous finding of heroin-induced upregulation of *circGrin2b* regulation [32]. Following 24 h morphine exposure, a one-way ANOVA indicated that morphine significantly increased expression of *circGrin2b*, with significant post hoc analyses for elevation of *circGrin2b* following both 0.1 µM and 10 µM morphine concentrations versus vehicle treatment (one-way ANOVA: F (3, 43)= 3.736, *p* = 0.018, Dunnett’s post hoc tests: 0.1 µM morphine vs. vehicle *p* = 0.0235; 10 µM vs. vehicle *p* = 0.0413; Figure 2A). These effects were specific for *circGrin2b*, as no significant regulation of linear *Grin2b* was observed in cortical cultures at any dose of morphine after 24 h exposure (Kruskal–Wallis test *p* = 0.0777; Figure 2B). Conversely, acute 90 min morphine exposure downregulated expression of both circ- and linear *Grin2b* (one-way ANOVA, *circGrin2b* 90 min: F (3, 55) = 3.198, *p* = 0.0304; one-way ANOVA linear *Grin2b* 90 min: F (3, 55)= 3.226, *p* = 0.0294; Figure 2C,D). Post hoc analyses of the 90 min timepoint revealed significant downregulation of *circGrin2b* following treatment with 1 µM morphine and linear *Grin2b* following treatment with both 1 µM and 10 µM morphine (Dunnett’s post hoc tests: *circGrin2b*: 1 µM morphine vs. vehicle, *p* = 0.0093; linear *Grin2b*: 1 µM morphine vs. vehicle, *p* = 0.0139, 10 µM morphine vs. vehicle, *p* = 0.0466; Figure 2D). This data demonstrates that morphine exposure dynamically regulates *circGrin2b* in opposing directions at 90 min and 24 h; and that morphine-induced upregulation of *circGrin2b* at 24 h is independent of the linear transcript.

### 2.3. circGrin2b Sponges miR-26b-3p

**Figure 3:**
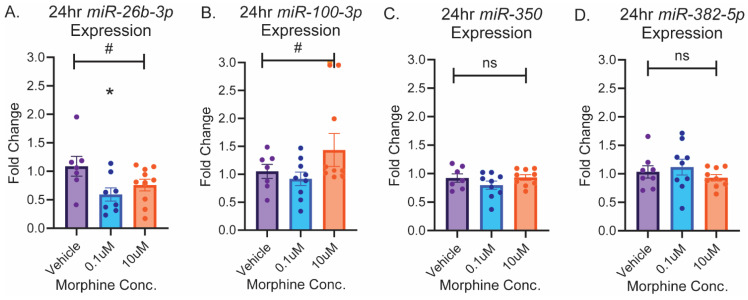
Morphine exposure regulates expression of miRNAs with putative *circGrin2b* binding sites. miRNA expression in primary cortical cultures following morphine treatment for *miR-26b-3p* (**A**), *miR-100-3p* (**B**), *miR-350* (**C**), and *miR-382-5p* (**D**). Error bars indicate the standard error of the mean (SEM). # above the solid line denotes one-way ANOVA *p* < 0.1 but *p* > 0.05; * directly above histogram indicate *p* < 0.05 versus vehicle post hoc test. ns, not significant.

We next sought to characterize the downstream consequences of opioid-induced *circGrin2b* regulation by exploring putative miRNA sponge targets. Our previously published microarray data set identified 5 putative miRNA sponge targets of *circGrin2b*: *miR-26b-3p*, *miR-100-3p*, *miR-350*, and *miR-382-5p*, and *miR-463-3p* (Supplementary Figure S2) [32]. *miR-463-3p* expression was undetectable in frontal cortex samples and therefore we excluded it from our analysis. The *circGrin2b* sequence is a perfect 7mer-m8 match for the seed sequences of *miR-26b-3p* and *miR-100-3p*, while *circGrin2b* is an 8mer match for *miR-350* and an imperfect match for *miR-382-5p* (Supplementary Figure S2). We measured expressions of these 4 putative target miRNAs in morphine-treated primary cortical cultures at time-points and concentrations that significantly upregulated *circGrin2b* compared to vehicle: 24 h, 0.1 µM, and 10 µM. We hypothesized that the levels of miRNAs that bind *circGrin2b* would be reduced compared to the upregulation of *circGrin2b* observed at 24 h post-morphine exposure, due to *circGrin2b* sponge activity. A strong trend for negative regulation of *miR-26b-3p* was observed following 24 h morphine exposure, with post hoc analyses showing a significant decrease in *miR-26b-3p* expression in the 0.1 µM concentration compared to vehicle (Kruskal–Wallis *p* = 0.0587; Dunnett’s post-hoc test: 0.1 µM vs. vehicle, *p* = 0.0372; Figure 3A). Trends for morphine-induced dysregulation of *miR-100-3p* were observed at 24 h, but they did not reach statistical significance (one-way ANOVA, 24 h morphine: F (2, 22) = 3.084, *p* = 0.0659; Figure 3B). Levels of *miR-350* and *miR-382-5p* were unchanged following 24 h morphine (Figure 3C,D).

**Figure 4:**
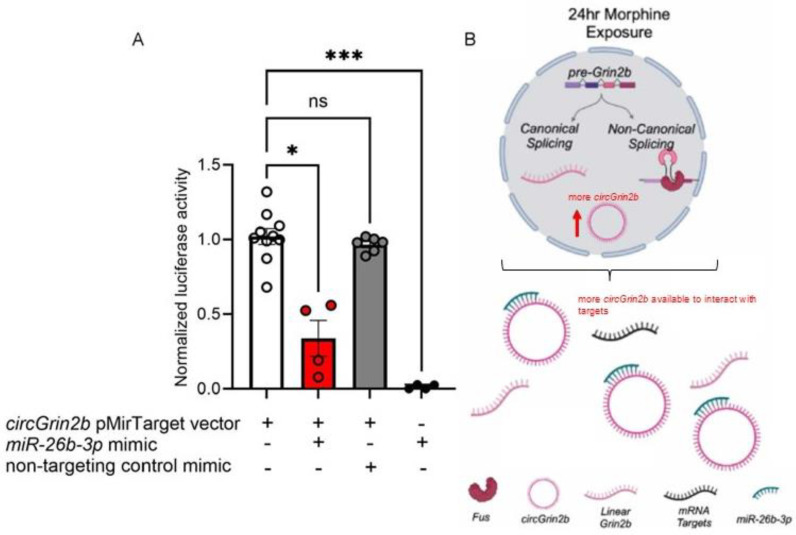
*circGrin2b* sponges *miR-26b-3p*. (**A**) Relative luminescence values in HEK293T cells following co-transfection of *circGrin2b* pMirTarget vector, a *miR-26b-3p* mimic, or a non-targeting control mimic. Mimics alone were used as a negative control and did not produce chemiluminescence. (**B**) Proposed mechanisms for *circGrin2b*-mediated opioid-induced neuroadaptations. Following 24 h morphine exposure, an increase in *circGrin2b* expression results in an elevation of *circGrin2b* available to interact with other targets, such as *miR-26b-3p*. * *p* < 0.05, *** *p* < 0.001, ns, not significant.

Based on the observed expression pattern of *miR-26b-3p* following morphine exposure, we evaluated the interaction between *circGrin2b* and *miR-26b-3p* using a luciferase reporter assay. The full-length *circGrin2b* sequence was subcloned into the pMirTarget luciferase reporter vector and HEK293T cells were transfected with the *circGrin2b* pMirTarget with a *miR-26b-3p* mimic or a non-targeting control mimic. We detected luminescence in HEK293T cells transfected with the *circGrin2b* luciferase reporter vector but a significant reduction in luminescence in samples co-transfected with *circGrin2b* pMirTarget vector and a *miR-26b-3p* mimic, while transfection with mimics alone yielded no luminescence signal (Kruskal–Wallis: *p* = 0.0007; Dunnet’s post-hoc tests: *circGrin2b* pMirTarget vector vs. *circGrin2b* pMirTarget vector + *miR-26b-3p* mimic *p* = 0.0008; Figure 4A). The nontargeting control mimic + *circGrin2b* pMirTarget vector was not different from the *circGrin2b* pMirTarget vector alone. This data suggests that when opioids regulate the expression of *circGrin2b*, there is opposing regulation of *miR-26b-3p*, and *circGrin2b* may functionally associate with *miR-26b-3p* (Figure 4B).

### 2.4. OFC circGrin2b Levels Are Negatively Correlated with miR-26b-3p Expression Following Heroin SA

**Figure 5:**
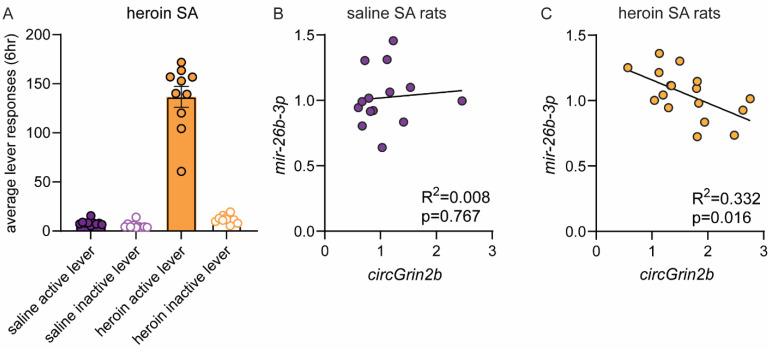
*circGrin2b* is negatively correlated with *miR-26b-3p* levels in the rat frontal cortex following opioid self-administration. (**A**) The average lever responses on the active lever paired with saline or heroin infusions, or an inactive lever during SA. Each dot represents the average lever responses for one day of SA. (**B**,**C**) Correlation between OFC *circGrin2b* expression with the putative miRNA target *miR-26b-3p* in rats that self-administered saline (**B**) or heroin (**C**).

To determine if the observed in vitro relationship between *circGrin2b* and *miR-26b-3p* occurs in the mammalian brain, we returned to our animal model of heroin SA. We measured expression of *miR-26b-3p* in OFC tissue samples from male and female rats that underwent heroin SA, as we previously reported that *circGrin2b* was significantly upregulated in these samples [32]. Rats that received heroin quickly learned to distinguish between a drug-paired active lever, and an inactive lever that did not result in any programmed consequences (Figure 5A). Rats that received saline during SA did not distinguish between the two levers. Levels of *circGrin2b* were not correlated with *miR-26b-3p* in rats that self-administered saline (Figure 5B). Conversely, rats that self-administered heroin displayed a significant negative correlation between OFC expression of *circGrin2b* and *miR-26b-3p* (Pearson’s Correlation, R^2^ = 0.3320, *p* = 0.0155; Figure 5C). This data demonstrates that the in vitro relationship we reported is consistent and relevant within an in vivo model of opioid-exposure.

## 3. Discussion

Our previous work was the first to characterize heroin-induced dysregulation of circRNAs [32]. The current study sought to further characterize the molecular impact of opioid exposure of one of these heroin-associated circRNAs, *circGrin2b*, which we selected to study because the parent gene, *Grin2b*, is essential for opioid learning and memory [14,48,49,50,51]. Our work has extended the current literature on the known impacts of *Grin2b* on opioid-induced phenotypes by reporting novel regulation of a splice variant of *Grin2b* following opioid exposure. Furthermore, we report dynamic regulation of *circGrin2b* after morphine exposure and have established a timeline for future studies to model opioid-induced *circGrin2b* dysregulation to perform mechanistic studies of *circGrin2b* regulatory function. We used this in vitro model to delineate potential splicing factors and downstream effectors of this novel circRNA species. 90 min morphine exposure induced a downregulation of both linear and *circGrin2b*; while 24 h morphine exposure specifically increased expression of *circGrin2b*. Our findings are supported by the existing linear *Grin2b* literature which shows dynamic opioid-induced regulation of *Grin2b* expression in the frontal cortex in both preclinical rat models and human subjects that misused opioids [32,57]. One inherent characteristic of circRNA expression is that it is dependent on the transcription of its linear counterpart. As such, it is interesting that we reported that morphine exposure induced a significant upregulation of *circGrin2b* while there was only a trend toward upregulation of linear *Grin2b* at 24 h morphine exposure. This demonstrates that opioid-induced *circGrin2b* dysregulation is not merely a byproduct of linear *Grin2b* regulation and suggests morphine exposure could preferentially induce biogenesis of *circGrin2b* in the absence of regulation of the linear parent gene.

To elucidate the mechanisms mediating *circGrin2b* biogenesis, we examined the expression of three putative splicing factors in morphine-exposed cortical cultures: *Fus*, *Adar1*, and *Qki*. We reported morphine-induced regulation of *Fus* and we hypothesize that *Fus* may be a potential splicing factor involved in *circGrin2b* biogenesis. *Fus* was found to be significantly downregulated at 90 min, specifically after the 1 µM morphine treatment. Similarly, at this timepoint and concentration, we also observed a decrease in expression of *circGrin2b*. At 24 h there was significant downregulation of *Fus* at the 0.1 µM morphine concentration, while this timepoint and concentration elicited significant upregulation of *circGrin2b*. *Fus* promotes circularization via complementary binding to intronic sequences, however published studies indicate that *Fus* negatively regulates circRNA biogenesis [52,56]. Thus, it is possible that *Fus* downregulation at 24 h negatively relieves inhibition of *circGrin2b*, resulting in upregulation. However, for the 90 min timepoint, it is unclear if downregulation of *Fus* mRNA within this short timeframe would result in meaningful reduction of Fus protein and further studies will be required to answer this question. For *circGrin2b* levels to be reduced within 90 min, degradation of the circRNA may occur. For these reasons, we believe that the significance of *circGrin2b* upregulation at 24 h is likely more analogous to the changes observed in the OFC following longer heroin exposure in our in vivo model.

Regarding the differential effects of *circGrin2b* regulation at 90 min versus 24 h, *Fus* expression could play a role in the observed changes but further studies in the future will be required to elucidate the relationship between *circGrin2b* and *Fus*. A noted limitation of the current study is the lack of mu-opioid receptor antagonists to demonstrate that blockade of morphine’s effects prevents regulation of *Fus*. In regard to the lack of dose-dependence in our treatment regimen, we speculate that one explanation of the differences in regulation of *circGrin2b* between doses may be desensitization of the mu-opioid receptor, or receptor internalization of the mu-opioid receptor at higher doses of morphine that result in effects at the 10 µM dose mirroring the 0.1 µM dose. While these findings are exciting, further research into the relationship between *Fus* and *circGrin2b* is necessary to delineate specific interactions. Future studies will explore the protein expression of Fus in cortical cells following morphine exposure to determine how meaningful morphine-induced regulation of *Fus* may translate into an impact on circRNA biogenesis. Additionally, published studies indicate that *Fus* may function as a regulator of circRNA biogenesis for many other circRNAs beyond *circGrin2b* [52,56,58]. In our prior published study, we identified 76 circRNAs regulated in the frontal cortex that result from chronic heroin SA in rats [32]. Whether *Fus* may regulate any other opioid-induced circRNAs following chronic opioid exposure is currently unknown. However, determining the specificity of *Fus*-induced regulation of *circGrin2b* following opioid exposure is a future goal that may elucidate a novel pathway of opioid-induced neuroadaptations in the brain.

Finally, this study sought to elucidate the downstream effects of *circGrin2b* regulation. Based on expression and correlation analyses, we selected *miR-26b-3p* as a putative miRNA candidate for functional interaction analysis using a luciferase assay. Our findings demonstrate that *circGrin2b* can functionally bind *miR-26b-3p* in vitro and are consistent with *circGrin2b* functioning as a miRNA sponge. To better understand the functional role of *circGrin2b*, understanding the mechanisms of *miR-26b-3p* is paramount. As a potential miRNA sponge, *circGrin2b* would bind to and sequester miRNA targets, thus facilitating translation of miRNA-repressed genes. *miR-26b-3p* is a member of the miR-26 family, which are highly expressed in neural tissues and are involved in neural progenitor cell (NPC) differentiation [59,60]. *miR-26b-3p* is dysregulated in several neuropsychiatric disorders, including fetal alcohol syndrome and Alzheimer’s Disease (AD) [61,62]. *miR-26b-3p* has been implicated in a myriad of molecular processes involved in cognition and synaptic plasticity [62]. For instance, *miR-26b-3p* has been found to: 1. negatively regulate neurite outgrowth, though the molecular mechanism is currently unknown [62]; 2. bind and suppress translation of brain-derived neurotrophic factor (BDNF), a protein vital to learning, memory, and higher thought processing [63,64]; 3. indirectly suppress the transcription factor (TF) nuclear factor-κB (NFκB) through intermediaries Tak1 and Tab3 [65]. As such, sponging of *miR-26b-3p* by *circGrin2b* could promote drug-induced neuroplasticity. Furthermore, indirect suppression of NFκB by *miR-26b-3p* is relevant as NFκB facilitates transcription of multiple drug-associated genes. Most intriguingly, *miR-26b-3p* is predicted to bind linear *Grin2b* with seven potential binding sites in the 3′ UTR according to Targetscan [66]. Thus, it is plausible to hypothesize that through sponging of *miR-26b-3p*, *circGrin2b* could facilitate both transcription (via NFκB) and translation of *Grin2b*; impact BDNF signaling vital to drug-associated behavioral phenotypes; and promote neurite outgrowth through sponging out *miR-26b-3p*.

## 4. Materials and Methods

### 4.1. Animals

Timed-pregnant female Sprague Dawley rats and adult male and female Sprague Dawley rats were purchased from Charles River Laboratory (Wilmington, MA, USA). Animals were fed standard chow ad libitum and housed at constant temperature (22 ± 2 °C) and humidity (40%) on a 12 h light/dark cycle. All procedures involving animals followed the National Institutes of Health’s Guide for the Care and Use of Laboratory Animals and were approved by Temple University’s Institutional Animal Care and Use Committee (IACUC). The sex of all rat pups and adult rats used in this study included both male and female rats. In vivo heroin SA procedures and presentation of SA data were previously reported in our prior publication [32]. Briefly, adult male and female rats self-administered 0.03 mg/kg/infusion of heroin in 6 h daily sessions for 10 days. Immediately after the last heroin session, rats were euthanized with isoflurane anesthesia, followed by rapid decapitation. Brains were removed from rats and frozen in liquid isopentane on dry ice. The OFC was dissected from each rat on a cold plate and dry ice.

### 4.2. Reagents

Morphine and diamorphine hydrochloride (heroin) were supplied by the National Institute on Drug Abuse drug supply program (Research Triangle Institute). Morphine was dissolved in primary culture media and heroin was dissolved in 0.9% sterile saline solution for SA.

### 4.3. Primary Frontal Cortex Cultures

Following gestation, newly born male and female rat pups were rapidly decapitated on post-natal day 1 (PN1). Brains were removed from skulls while submerged in Hank’s Buffered Salt Solution (HBSS) with the aid of a dissecting microscope. Frontal cortices were isolated and resuspended at a density of ~500,000/well on poly-d-lysine-coated (Gibco, Fredrick, MD, USA) 6-well plates (Corning Incorporated, Corning, NY, USA) in culture medium containing Neurobasal-A Medium 1× (Gibco), 1× B27 supplement (Gibco), 2 mM L-glutamine (Gibco), and 1% Penicillin-streptomycin (Gibco). Cortical cultures were incubated at 37 °C in an Isotemp CO_2_ Incubator (FisherBrand, Waltham, MA, USA) and cultured for 5 days in vitro (DIV) before treatment. Cells were treated with morphine dissolved in pre-warmed culture media to concentrations of 0.1 µM, 1 µM, and 10 µM. Cells were collected on DIV6.

### 4.4. RNA Extraction

To harvest primary cortical cultures for RNA isolation, cell culture media was aspirated and wells were washed in 1× sterile PBS. Cells were scraped from the wells in 200 µL of ice-cold CD buffer from the MIRvana Paris Protein & RNA Isolation System (ThermoFisher Scientific, Waltham, MA, USA). An additional 50–60 µL of CD buffer was used to wash wells. Cells were collected and frozen at −80 °C until RNA was extracted. For extraction of RNA from OFC tissue samples of rats following heroin SA, bilateral tissue punches from the OFC were homogenized in 250 µL of CD buffer. Total RNA was extracted using the MIRvana Paris Protein & RNA Isolation System (ThermoFisher Scientific) according to the manufacturer’s instructions, as we have previously reported [67], and RNA concentration was measured using a Qubit (Invitrogen, Waltham, MA, USA).

### 4.5. Gene Expression Assays

Quantitative polymerase chain reaction (qPCR) assays for measurement of gene expression were performed on a Quantstudio3 qPCR Machine (ThermoFisher Scientific). To measure circRNAs and mRNAs, cDNA was synthesized with ~250ng of total RNA and was reverse transcribed using the Maxima Reverse Transcriptase (ThermoFisher Scientific) according to manufacturer’s protocols. cDNA, diluted 1:3 with RNAse free water, was used as a template in qPCR reactions. circRNAs and their linear counterparts were measured with IDT PrimeTime Gene Expression MasterMix and IDT PrimeTime qPCR Probe Assays (Integrated DNA Technologies, Coralville, IA, USA). Biogenesis enzyme mRNAs were measured with PerfeCTa Fast Mix II (QuantaBio, Beverly, MA, USA) and TaqMan Gene Expression Primers (ThermoFisher Scientific). Glyceraldehyde 3-phosphate dehydrogenase (*Gapdh*) and β-actin (*Actb*) were used as housekeeping genes for normalization of circRNA and mRNA expressions. For measurement of miRNA expression, cDNA was synthesized from 100 ng of total RNA using the miRCURY LNA RT kit (Qiagen, Germantown, MD, USA), as previously described [68]. miRNA expression was quantified with qPCR using the miRCURY LNA SYBR Green PCR Master Mix and LNA Primers (Qiagen, Germantown, MD, USA). miRNA expression was normalized to *rno-miR-320a*, a miRNA that displays minimal response to opioid exposure, as we previously reported [69]. Expression levels were calculated using the delta–delta CT method [70]. A full list of primers can be found in Supplementary Table S1.

### 4.6. circRNA Primer Validation

*circGrin2b_011731* qPCR product size was validated with agarose gel electrophoresis. Briefly, PCR was performed as outlined above and the product was collected. Agarose (Fisher Scientific, Waltham, MA, USA) was dissolved in 1× Tris-Borate EDTA (TBE) Buffer (Fisher Scientific) for a final concentration of 1% agarose and heated in 30 s intervals until solution was clear. The solution was allowed to cool slightly before Sybr Safe (Invitrogen, Waltham, MA, USA) was added for a final ratio of 1 µL/100 mL 1× TBE. The solution was added to Owl EasyCast B2 Gel Electrophoresis Systems (Fisher Scientific) apparatus and allowed to solidify. Samples were combined with 6× DNA loading dye (Thermo Scientific, Waltham, MA, USA) and run alongside a 100 bp DNA Ladder (Invitrogen, Waltham, MA, USA) on the gel for 2 h at 70 volts. Gels were imaged using a Fujifilm LAS-1000 Intelligent Dark Box II machine under UV light (Fujifilm, Tokyo, Japan).

Further confirmation of PCR amplification of the back-splice junction (BSJ) of endogenous *circGrin2b* was determined by Sanger Sequencing. qPCR products obtained were subcloned into the TOPO Cloning Vector (ThermoFisher Scientific) according to the manufacturer’s specifications, as we have previously described [21]. Sanger Sequencing was performed on DNA by Genewiz (Azenta Life Sciences, South Plainfield, NJ, USA). The sequencing results were compared to the *Grin2b* sequence obtained from the rat genome (version rn5) obtained from the UCSC Genome Browser (Supplementary Figure S1). As we previously described, Rnase R treatment was additionally used to demonstrate that *circGrin2b* primers amplify a product resistant to Rnase R degradation, while linear *Grin2b* primers do not [32].

### 4.7. Luciferase Reporter Assay

Potential *circGrin2b*-miRNA interactions were examined in vitro using a luciferase reporter assay [71], with modification. HEK-293T cells were cultured in Dulbecco’s Modified Eagle Medium (DMEM) with 10% fetal bovine serum (FBS) and 1% penicillin/streptomycin (pen/strep) media. HEK293T cells were co-transfected with a pMirTarget 3′UTR reporter vector containing the full 599 nt sequence of *circGrin2b* downstream of the firefly luciferase reporter (Origene, Rockville, MD, USA) and miRIDIAN miRNA mimics for *hsa-miR-26b-3p* (Horizon Discovery, Cambridge, UK) using Lipofectomine 3000 transfection reagent (Invitrogen, Waltham, MA, USA) and serum free-DMEM, according to manufacturer’s protocols. Media was aspirated 2 h after transfection and replaced with fresh DMEM media containing 10% FBS-1% pen/strep. 48 h after transfection, cells were harvested using the Luciferase Cell Culture Lysis 5× Reagent (Promega, Madison, WI, USA). Briefly, media was removed and cells were washed once with 1× PBS. 100 µL of 1× Lysis Reagent was added to each well and trays were rocked gently for ~30 s. A cell scraper was used to detach cells from the plate. Cell lysates were spun at 12,000 G for 15 s and supernatant was collected and stored at −80 °C. Luminescence was measured using the Luciferase Reporter Assay System (Promega, Madison, WI, USA) according to the manufacturer’s instructions, and quantified with an Infinite M200Pro plate reader (Tecan, Zürich, Switzerland).

### 4.8. Statistics

Data are presented as the mean, with error bars indicating the standard error of the mean (SEM). D’Agostino normality tests were performed on all data sets. One-way analysis of variance (ANOVA) with Dunnet’s multiple comparisons post hoc tests were performed on all datasets with a normal distribution. For datasets without a normal distribution, Kruskal–Wallis tests with Dunn’s multiple comparisons post hoc tests were performed. Unpaired Student’s *t*-tests were used to analyze the mean variance between two groups with normally distribution. qPCR statistical analyses were performed on the delta-delta CT values prior to log transformation.

## 5. Conclusions

In summary, the current study reports that opioids regulate expression of *circGrin2b*, and a potential circRNA biogenesis factor, *Fus*, in frontal cortex cells. Cortical *circGrin2b* regulation is an opioid-induced neuroadaptation and *circGrin2b* interacts with *miR-26b-3p*, a miRNA which regulates synaptic plasticity and drug-associated learning.

## Data Availability

Data presented in this article are available from the authors upon a reasonable request.

## Supplementary Materials

The following supporting information can be downloaded at: https://www.mdpi.com/article/10.3390/ijms26115010/s1.

## References

[1] Ahmad F.B., Cisewski J.A., Rossen L.M., Sutton P. (2022). National Center for Health Statistics. https://www.cdc.gov/nchs/nvss/vsrr/drug-overdose-data.htm.

[2] Centers for Disease Control and Prevention Multiple Cause of Death Files, National Center for Health Statistics. 1999–2019. https://wonder.cdc.gov/.

[3] Hedegaard H., Miniño A.M., Warner M. (2021). Co-involvement of opioids in drug overdose deaths involving cocaine and psychostimulants. NCHS Data Briefs.

[4] Volkow N.D., Morales M. (2015). The Brain on Drugs: From Reward to Addiction. Cell.

[5] Smyth B.P., Barry J., Keenan E., Ducray K. (2010). Lapse and relapse following inpatient treatment of opiate dependence. Ir. Med. J..

[6] Grella C.E., Lovinger K. (2011). 30-year trajectories of heroin and other drug use among men and women sampled from methadone treatment in California. Drug Alcohol Depend..

[7] Chalana H., Kundal T., Gupta V., Malhari A.S. (2016). Predictors of Relapse after Inpatient Opioid Detoxification during 1-Year Follow-Up. J. Addict..

[8] Madsen H.B., Brown R.M., Short J.L., Lawrence A.J. (2012). Investigation of the neuroanatomical substrates of reward seeking following protracted abstinence in mice. J. Physiol..

[9] Lou M., Wang E., Shen Y., Wang J. (2012). Cue-elicited craving in heroin addicts at different abstinent time: An fMRI pilot study. Subst. Use Misuse.

[10] Christie M.J. (2008). Cellular neuroadaptations to chronic opioids: Tolerance, withdrawal and addiction. Br. J. Pharmacol..

[11] Gradin V.B., Baldacchino A., Balfour D., Matthews K., Steele J.D. (2014). Abnormal brain activity during a reward and loss task in opiate-dependent patients receiving methadone maintenance therapy. Neuropsychopharmacology.

[12] Kauer J.A., Malenka R.C. (2007). Synaptic plasticity and addiction. Nat. Rev. Neurosci..

[13] Kruyer A., Chioma V.C., Kalivas P.W. (2020). The Opioid-Addicted Tetrapartite Synapse. Biol. Psychiatry.

[14] Shen H., Moussawi K., Zhou W., Toda S., Kalivas P.W. (2011). Heroin relapse requires long-term potentiation-like plasticity mediated by NMDA2b-containing receptors. Proc. Natl. Acad. Sci. USA.

[15] Bell J., Strang J. (2020). Medication Treatment of Opioid Use Disorder. Biol. Psychiatry.

[16] You X., Vlatkovic I., Babic A., Will T., Epstein I., Tushev G., Akbalik G., Wang M., Glock C., Quedenau C. (2015). Neural circular RNAs are derived from synaptic genes and regulated by development and plasticity. Nat. Neurosci..

[17] Barrett S.P., Salzman J. (2016). Circular RNAs: Analysis, expression and potential functions. Development.

[18] Kristensen L.S., Andersen M.S., Stagsted L.V.W., Ebbesen K.K., Hansen T.B., Kjems J. (2019). The biogenesis, biology and characterization of circular RNAs. Nat. Rev. Genet..

[19] Li X., Yang L., Chen L.L. (2018). The Biogenesis, Functions, and Challenges of Circular RNAs. Mol. Cell.

[20] Bai X., Huang Y., Zhang K., Huang W., Mu Y., Li Y., Ouyang H. (2023). CircNf1-mediated CXCL12 expression in the spinal cord contributes to morphine analgesic tolerance. Brain Behav. Immun..

[21] Dabrowski K.R., Floris G., Gillespie A., Daws S.E. (2023). Orbitofrontal intronic circular RNA from Nrxn3 mediates reward learning and motivation for reward. Prog. Neurobiol..

[22] Hafez A.K., Zimmerman A.J., Papageorgiou G., Chandrasekaran J., Amoah S.K., Lin R., Lozano E., Pierotti C., Dell’Orco M., Hartley B.J. (2022). A bidirectional competitive interaction between circHomer1 and Homer1b within the orbitofrontal cortex regulates reversal learning. Cell Rep..

[23] Lu Y., Tan L., Wang X. (2019). Circular HDAC9/microRNA-138/Sirtuin-1 Pathway Mediates Synaptic and Amyloid Precursor Protein Processing Deficits in Alzheimer’s Disease. Neurosci. Bull..

[24] Piwecka M., Glažar P., Hernandez-Miranda L.R., Memczak S., Wolf S.A., Rybak-Wolf A., Filipchyk A., Klironomos F., Cerda Jara C.A., Fenske P. (2017). Loss of a mammalian circular RNA locus causes miRNA deregulation and affects brain function. Science.

[25] Shen Q., Xie B., Galaj E., Yu H., Li X., Lu Y., Zhang M., Wen D., Ma C. (2022). CircTmeff-1 in the nucleus accumbens regulates the reconsolidation of cocaine-associated memory. Brain Res. Bull..

[26] Zhang Y., Du L., Bai Y., Han B., He C., Gong L., Huang R., Shen L., Chao J., Liu P. (2020). CircDYM ameliorates depressive-like behavior by targeting miR-9 to regulate microglial activation via HSP90 ubiquitination. Mol. Psychiatry.

[27] Zimmerman A.J., Hafez A.K., Amoah S.K., Rodriguez B.A., Dell’Orco M., Lozano E., Hartley B.J., Alural B., Lalonde J., Chander P. (2020). A psychiatric disease-related circular RNA controls synaptic gene expression and cognition. Mol. Psychiatry.

[28] Jeck W.R., Sharpless N.E. (2014). Detecting and characterizing circular RNAs. Nat. Biotechnol..

[29] Jeck W.R., Sorrentino J.A., Wang K., Slevin M.K., Burd C.E., Liu J., Marzluff W.F., Sharpless N.E. (2013). Circular RNAs are abundant, conserved, and associated with ALU repeats. RNA.

[30] Ragan C., Goodall G.J., Shirokikh N.E., Preiss T. (2019). Insights into the biogenesis and potential functions of exonic circular RNA. Sci. Rep..

[31] Rybak-Wolf A., Stottmeister C., Glažar P., Jens M., Pino N., Giusti S., Hanan M., Behm M., Bartok O., Ashwal-Fluss R. (2015). Circular RNAs in the Mammalian Brain Are Highly Abundant, Conserved, and Dynamically Expressed. Mol. Cell.

[32] Floris G., Gillespie A., Zanda M.T., Dabrowski K.R., Daws S.E. (2022). Heroin Regulates Orbitofrontal Circular RNAs. Int. J. Mol. Sci..

[33] Paudel P., Pierotti C., Lozano E., Amoah S.K., Gardiner A.S., Caldwell K.K., Allan A.M., Mellios N. (2020). Prenatal Alcohol Exposure Results in Sex-Specific Alterations in Circular RNA Expression in the Developing Mouse Brain. Front. Neurosci..

[34] Daws S.E., Gillespie A. (2023). Circular RNA regulation and function in drug seeking phenotypes. Mol. Cell. Neurosci..

[35] Dube U., Del-Aguila J.L., Li Z., Budde J.P., Jiang S., Hsu S., Ibanez L., Fernandez M.V., Farias F., Norton J. (2019). An atlas of cortical circular RNA expression in Alzheimer disease brains demonstrates clinical and pathological associations. Nat. Neurosci..

[36] Mahmoudi E., Cairns M.J. (2019). Circular RNAs are temporospatially regulated throughout development and ageing in the rat. Sci. Rep..

[37] Zajaczkowski E.L., Bredy T.W. (2021). Circular RNAs in the Brain: A Possible Role in Memory?. Neuroscientist.

[38] Vornholt E., Drake J., Mamdani M., McMichael G., Taylor Z.N., Bacanu S.A., Miles M.F., Vladimirov V.I. (2021). Identifying a novel biological mechanism for alcohol addiction associated with circRNA networks acting as potential miRNA sponges. Addict. Biol..

[39] Chen Y., Li X., Meng S., Huang S., Chang S., Shi J. (2022). Identification of Functional CircRNA-miRNA-mRNA Regulatory Network in Dorsolateral Prefrontal Cortex Neurons of Patients With Cocaine Use Disorder. Front. Mol. Neurosci..

[40] Boroujeni M.E., Nasrollahi A., Boroujeni P.B., Fadaeifathabadi F., Farhadieh M., Tehrani A.M., Nakhaei H., Sajedian A.M., Peirouvi T., Aliaghaei A. (2020). Exposure to methamphetamine exacerbates motor activities and alters circular RNA profile of cerebellum. J. Pharmacol. Sci..

[41] Bu Q., Long H., Shao X., Gu H., Kong J., Luo L., Liu B., Guo W., Wang H., Tian J. (2019). Cocaine induces differential circular RNA expression in striatum. Transl. Psychiatry.

[42] Dell’Orco M., Elyaderani A., Vannan A., Sekar S., Powell G., Liang W.S., Neisewander J.L., Perrone-Bizzozero N.I. (2021). HuD Regulates mRNA-circRNA-miRNA Networks in the Mouse Striatum Linked to Neuronal Development and Drug Addiction. Biology.

[43] Liu Y., Li J., Bu H., Wang H., Zhang Y., Shen Q., Li M., Lu Z., Rong X., Zheng D. (2021). Circular RNA expression alteration identifies a novel circulating biomarker in serum exosomal for detection of alcohol dependence. Addict. Biol..

[44] Altshuler R.D., Yang E.S., Garcia K.T., Davis I.R., Olaniran A., Haile M., Razavi S., Li X. (2021). Role of orbitofrontal cortex in incubation of oxycodone craving in male rats. Addict. Biol..

[45] Fanous S., Goldart E.M., Theberge F.R., Bossert J.M., Shaham Y., Hope B.T. (2012). Role of orbitofrontal cortex neuronal ensembles in the expression of incubation of heroin craving. J. Neurosci..

[46] Reiner D.J., Lofaro O.M., Applebey S.V., Korah H., Venniro M., Cifani C., Bossert J.M., Shaham Y. (2020). Role of Projections between Piriform Cortex and Orbitofrontal Cortex in Relapse to Fentanyl Seeking after Palatable Food Choice-Induced Voluntary Abstinence. J. Neurosci..

[47] Schoenbaum G., Shaham Y. (2008). The role of orbitofrontal cortex in drug addiction: A review of preclinical studies. Biol. Psychiatry.

[48] LaLumiere R.T., Kalivas P.W. (2008). Glutamate release in the nucleus accumbens core is necessary for heroin seeking. J. Neurosci..

[49] Peters J., De Vries T.J. (2012). Glutamate mechanisms underlying opiate memories. Cold Spring Harb. Perspect. Med..

[50] Ma Y.Y., Yu P., Guo C.Y., Cui C.L. (2011). Effects of ifenprodil on morphine-induced conditioned place preference and spatial learning and memory in rats. Neurochem. Res..

[51] Narita M., Aoki T., Suzuki T. (2000). Molecular evidence for the involvement of NR2B subunit containing N-methyl-D-aspartate receptors in the development of morphine-induced place preference. Neuroscience.

[52] Errichelli L., Dini Modigliani S., Laneve P., Colantoni A., Legnini I., Capauto D., Rosa A., De Santis R., Scarfò R., Peruzzi G. (2017). FUS affects circular RNA expression in murine embryonic stem cell-derived motor neurons. Nat. Commun..

[53] Shi L., Yan P., Liang Y., Sun Y., Shen J., Zhou S., Lin H., Liang X., Cai X. (2017). Circular RNA expression is suppressed by androgen receptor (AR)-regulated adenosine deaminase that acts on RNA (ADAR1) in human hepatocellular carcinoma. Cell Death Dis..

[54] Conn S.J., Pillman K.A., Toubia J., Conn V.M., Salmanidis M., Phillips C.A., Roslan S., Schreiber A.W., Gregory P.A., Goodall G.J. (2015). The RNA binding protein quaking regulates formation of circRNAs. Cell.

[55] Omata Y., Okawa M., Haraguchi M., Tsuruta A., Matsunaga N., Koyanagi S., Ohdo S. (2022). RNA editing enzyme ADAR1 controls miR-381-3p-mediated expression of multidrug resistance protein MRP4 via regulation of circRNA in human renal cells. J. Biol. Chem..

[56] Li X., Liu C.X., Xue W., Zhang Y., Jiang S., Yin Q.F., Wei J., Yao R.W., Yang L., Chen L.L. (2017). Coordinated circRNA Biogenesis and Function with NF90/NF110 in Viral Infection. Mol. Cell.

[57] Daneshparvar H., Sadat-Shirazi M.S., Fekri M., Khalifeh S., Ziaie A., Esfahanizadeh N., Vousooghi N., Zarrindast M.R. (2019). NMDA receptor subunits change in the prefrontal cortex of pure-opioid and multi-drug abusers: A post-mortem study. Eur. Arch. Psychiatry Clin. Neurosci..

[58] Garikipati V.N.S., Verma S.K., Cheng Z., Liang D., Truongcao M.M., Cimini M., Yue Y., Huang G., Wang C., Benedict C. (2019). Circular RNA CircFndc3b modulates cardiac repair after myocardial infarction via FUS/VEGF-A axis. Nat. Commun..

[59] Dill H., Linder B., Fehr A., Fischer U. (2012). Intronic miR-26b controls neuronal differentiation by repressing its host transcript, ctdsp2. Genes Dev..

[60] Sauer M., Was N., Ziegenhals T., Wang X., Hafner M., Becker M., Fischer U. (2021). The miR-26 family regulates neural differentiation-associated microRNAs and mRNAs by directly targeting REST. J. Cell Sci..

[61] Stringer R.L., Laufer B.I., Kleiber M.L., Singh S.M. (2013). Reduced expression of brain cannabinoid receptor 1 (Cnr1) is coupled with an increased complementary micro-RNA (miR-26b) in a mouse model of fetal alcohol spectrum disorders. Clin. Epigenet..

[62] Chu T., Shu Y., Qu Y., Gao S., Zhang L. (2018). miR-26b inhibits total neurite outgrowth, promotes cells apoptosis and downregulates neprilysin in Alzheimer’s disease. Int. J. Clin. Exp. Pathol..

[63] Caputo V., Sinibaldi L., Fiorentino A., Parisi C., Catalanotto C., Pasini A., Cogoni C., Pizzuti A. (2011). Brain derived neurotrophic factor (BDNF) expression is regulated by microRNAs miR-26a and miR-26b allele-specific binding. PLoS ONE.

[64] Lu B., Nagappan G., Lu Y. (2014). BDNF and synaptic plasticity, cognitive function, and dysfunction. Handb. Exp. Pharmacol..

[65] Zhao N., Wang R., Zhou L., Zhu Y., Gong J., Zhuang S.M. (2014). MicroRNA-26b suppresses the NF-κB signaling and enhances the chemosensitivity of hepatocellular carcinoma cells by targeting TAK1 and TAB3. Mol. Cancer.

[66] Friedman R.C., Farh K.K., Burge C.B., Bartel D.P. (2009). Most mammalian mRNAs are conserved targets of microRNAs. Genome Res..

[67] Zanda M.T., Floris G., Daws S.E. (2023). Orbitofrontal cortex microRNAs support long-lasting heroin seeking behavior in male rats. Transl. Psychiatry.

[68] Gillespie A., Mayberry H.L., Wimmer M.E., Daws S.E. (2022). microRNA expression levels in the nucleus accumbens correlate with morphine-taking but not morphine-seeking behaviour in male rats. Eur. J. Neurosci..

[69] Zanda M.T., Saikali L., Morris P., Daws S.E. (2023). MicroRNA-mediated translational pathways are regulated in the orbitofrontal cortex and peripheral blood samples during acute abstinence from heroin self-administration. Adv. Drug Alcohol Res..

[70] Livak K.J., Schmittgen T.D. (2001). Analysis of relative gene expression data using real-time quantitative PCR and the 2(-Delta Delta C(T)) Method. Methods.

[71] Rom S., Dykstra H., Zuluaga-Ramirez V., Reichenbach N.L., Persidsky Y. (2015). miR-98 and let-7g* protect the blood-brain barrier under neuroinflammatory conditions. J. Cereb. Blood Flow Metab..

