# Supplementary material for: Opioid-Induced Regulation of Cortical Circular-Grin2b_011731 Is Associated with Regulation of circGrin2b Sponge Target miR-26b-3p
Source: Int J Mol Sci. 2025 May 22;26(11):5010. doi: 10.3390/ijms26115010 (PMC12154416; doi:10.3390/ijms26115010)

SUPPLEMENTARY TABLE S1: LIST OF qPCR PRIMERS Integrated DNA Technology		
rno_circGrin2b_011731 (IDT)	Probe	5'-/56-FAM/TGGAAGAAC/ZEN/ ATGGAGGACTCATCCCT /31ABkFQ/-3'
	Primer 1	5'-CAAGAGCAGTTGCT ACAACAC-3'
	Primer 2	5'-TTCGAT AGACGGGCCAAAC-3'
Linear *Grin2b* (IDT)	Probe	5'-/ 56-FAM/TCTGCCTTC/Z EN/TT AGAGCCA TTCAGCG /31ABkFQ/-3'
	Primer 1	5'-GCATCAGTGTCATGGTATCTCG-3'
	Primer 2	5'-CACAAACATCATCACCCACAC-3'
	Assay ID	Rn.PT.58.9183827
Gapdh (IDT)	Probe	5'-/ 56-FAM/CACACCGAC/Z EN/CTTCACCA TCTTGTCT /31ABkFQ/-3
	Primer 1	5'-TCTCTGCTCCTCCCTGTTC-3'
	Primer 2	5'-GT AACCAGGCGTCCGATAC-3'
	Assay ID	Rn.PT.58.35727291
Fus (ThermoFisher)	Assay ID	Rn01508191_g1
Adar1 (ThermoFisher)	Assay ID	Rn00508006_m1
Qki (ThermoFisher)	Assay ID	Mm00498991_m1
Eif4e (ThermoFisher)	Assay ID	Rn00821567_g1
Hnrnph2 (ThermoFisher)	Assay ID	Rn01474230_m1
Gapdh (ThermoFisher)	Assay ID	Rn01775763_g1
hsa-miR-320a-3p (Qiagen)	Sequence	MIMAT0000510: 5'AAAAGCUGGGUUGAGAGGGCGA
	Assay ID	YP00206042
mmu-miR-26b03p (Qiagen)	Sequence	MIMAT0004630: 5'CCUGUUCUCCAUUACUUGGCUC
	Assay ID	YP02127459
rno-miR-100-3p (Qiagen)	Sequence	MIMAT0017112: 5'CAAGCUUGUGUCUAUAGGU
	Assay ID	YP02104369
rno-miR-350 (Qiagen)	Sequence	MIMAT0000604: 5'UUCACAAAGCCCAUACACUUUCAC
	Assay ID	YP00205719
hsa-miR-382-5p (Qiagen)	Sequence	MIMAT0000737: 5'GAAGUUGUUCGUGGUGGAUUCG
	Assay ID	YP00204169

Supplemental Figure S1: *circGrin2b* is a circular RNA derived from linear *Grin2b*.

Schematic depicting the structure of *circGrin2b*_011731, which is derived from exon 3 of the linear transcript and contains 599bp. B. Clustal Sequence alignment validating the qPCR product is consistent with *circGrin2b*. Highlighted portions of the alignment correspond with PCR primers and probe. Primer 1 corresponds with the reserve complement.

A.

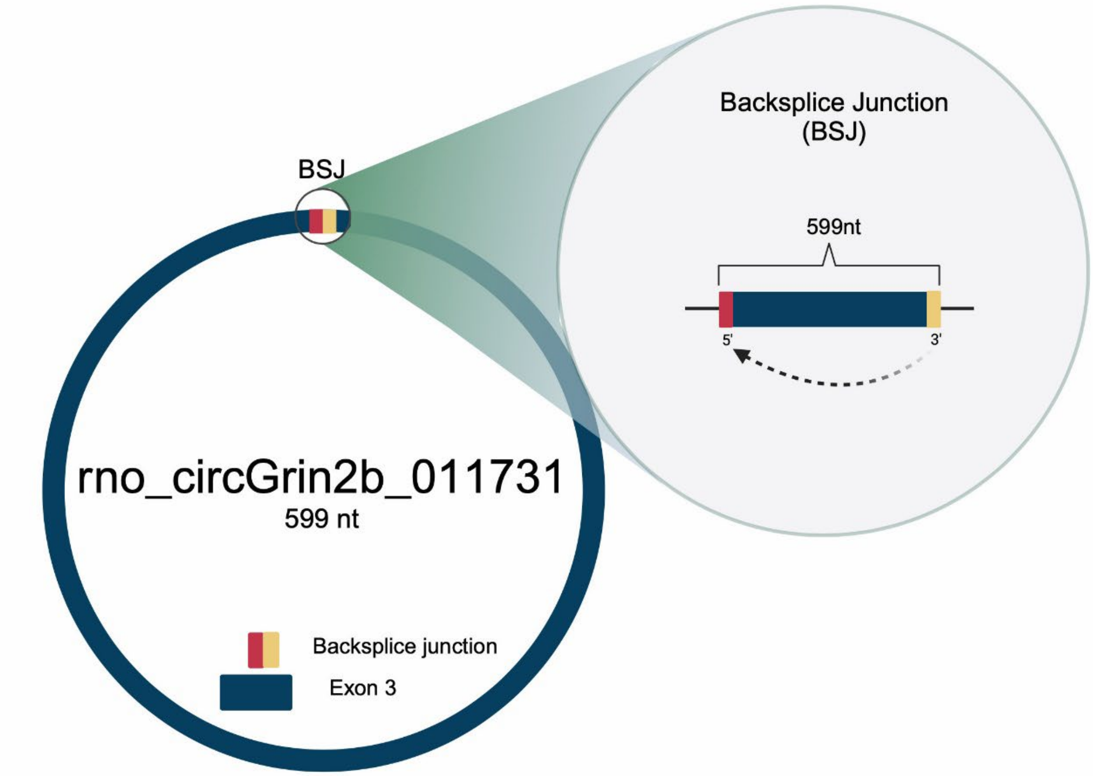

B.

```
CLUSTAL O(1.2.4) multiple sequence alignment


Grin2b_reference      TTCGATAGACGGGCCAAACTGGAAGAACATGGAGGACTCATCCTATTCAACATGTTAGAC
Grin2b_seq            TTCGATAGACGGGCCAAACTGGAAGAACATGGAGGACTCATCCTATTCAACATGTTAGAC
                      ************************************************************

Grin2b_reference      TGGTAGATCCTCTTCTCGTGGGTGTTGTAGCAACTGCTCTTG
Grin2b_seq            TGGTAGATCCTCTTCTCGTGGGTGTTGTAGCAACTGCTCTTG
                      ******************************************
Primer 1: CAAGAGCAGTTGCTACAACAC
Primer 2: TTCGATAGACGGGCCAAAC
Probe: TGGAAGAACATGGAGGACTCATCCCT
```

Supplemental Figure S2: Putative miRNA binding sites for circGrin2b. A-D. miRNA-*circGrin2b* binding sites for *miR-26b-3p* (A), *miR-100-3p* (B), *miR-350* (C), and *miR-382-5p* (D).

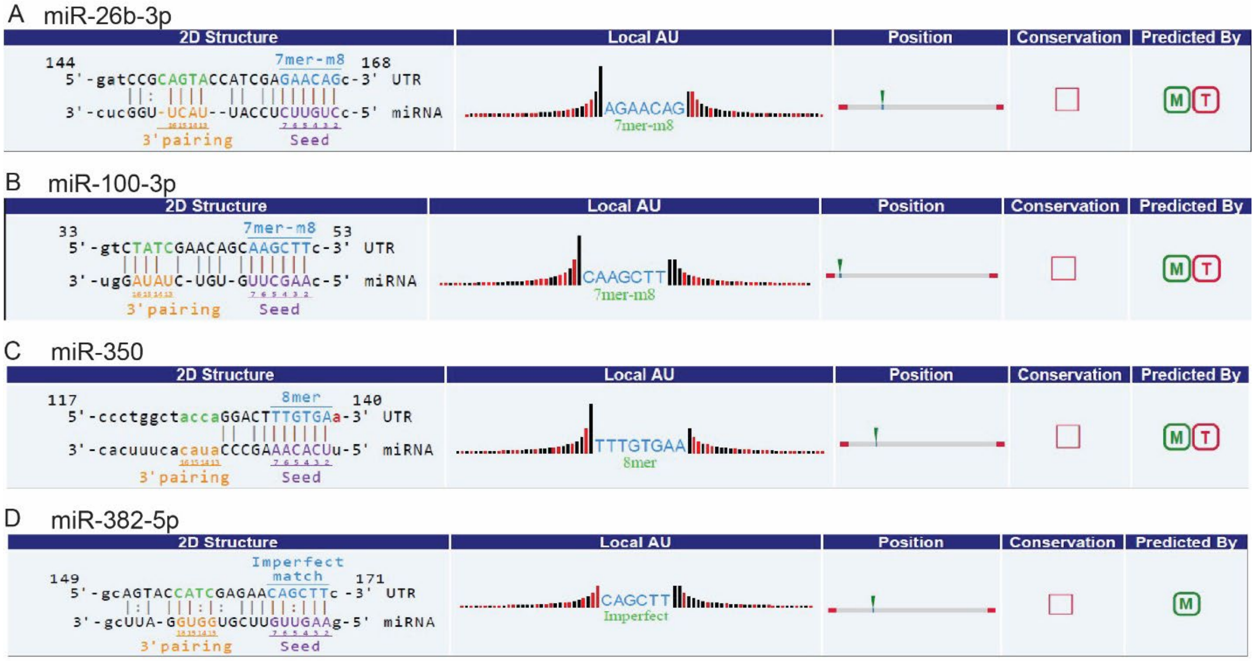

Supplement: Supplementary file 1 [file ijms-26-05010-s001.zip › ijms-3582838-supplementary.pdf]
